# A Reference-Free Lens-Flare-Aware Detector for Autonomous Driving

**DOI:** 10.3390/s26082359

**Published:** 2026-04-11

**Authors:** Shanxing Ma, Tim Willems, Wenwen Ma, Marwan Yusuf, David Van Hamme, Jan Aelterman, Wilfried Philips

**Affiliations:** Department of Telecommunications and Information Processing-Image Processing and Interpretation (TELIN-IPI), Ghent University—imec, Sint-Pietersnieuwstraat 41, 9000 Ghent, Belgium; tim.willems@ugent.be (T.W.); wenwen.ma@ugent.be (W.M.); marwan.yusuf@ugent.be (M.Y.); david.vanhamme@ugent.be (D.V.H.); jan.aelterman@ugent.be (J.A.); wilfried.philips@ugent.be (W.P.)

**Keywords:** autonomous driving, object detection, lens flare, likelihood ratio

## Abstract

As autonomous driving technology advances, the deployment of autonomous vehicles in urban environments is rapidly increasing. Lens flare—an often overlooked optical artifact in object detection research—can lead to increased false positives or missed detections, particularly in the challenging conditions inherent to autonomous driving. Current mitigation methods are often ill-suited for real-time implementation. This work proposes a solution to alleviate the adverse effects of lens flare by utilizing a lightweight lens flare perception network, eliminating the need for additional hardware or complex image pre-processing. Specifically, we propose a reference-free model utilizing a ResNet18 backbone integrated with a lightweight Multi-Layer Perceptron (MLP) to extract and leverage lens flare information. This model is developed via a teacher–student framework, which was distilled from an end-to-end reference-based model optimized using the Learned Perceptual Image Patch Similarity (LPIPS) metric. Our experiments demonstrate that incorporating lens flare information significantly enhances the performance of the baseline object detection network, outperforming previous mitigation methods by a substantial margin. The proposed method can be seamlessly integrated into existing object detectors and requires only an efficient training process, facilitating its deployment in practical autonomous driving tasks.

## 1. Introduction

Autonomous Driving Systems (ADSs) have advanced rapidly in recent years as a promising means of reducing human error and preventing road accidents [1]. To achieve higher levels of autonomy, numerous challenges must be addressed across various sub-tasks, including object detection and path planning. As a fundamental component, object detection is crucial to the overall performance of ADSs; however, it continues to face significant hurdles.

Cameras are widely utilized for object detection in ADSs because they provide rich contextual information, such as color and texture. However, as passive sensors, cameras only capture ambient light, making their performance highly sensitive to adverse environmental conditions, such as extreme lighting, fog, and rain [2]. In this work, we address the impact of lens flare—an optical artifact highlighted in the IEEE P2020 Automotive Imaging White Paper [3]—on object detection, a factor that has been largely overlooked in existing literature.

**Figure 1:**
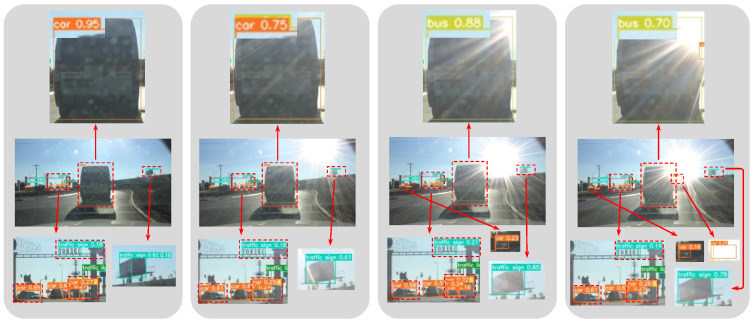
Impact of lens flare location on object detection performance. Detection results from a single detector applied to the same scene with lens flare appearing at varying positions. This comparison demonstrates that lens flare—regardless of its intensity—can fluctuate detection scores and degrade overall performance. Quantifying this degradation using a specialized metric facilitates the development of detection models with enhanced robustness to lens flare artifacts.

Lens flare exerts a significant influence on both object detector performance and overall perceptual quality [4]. As illustrated in Figure 1, lens flare—regardless of its intensity—can affect detection results in various ways, depending on factors such as its position, color, and shape. Pixel-wise metrics, such as Mean Squared Deviation (MSD), between perfectly aligned lens-flare-free and lens-flare-corrupted images can quantify flare intensity. However, these methods require access to a perfectly aligned, flare-free reference, limiting their practical applicability in ADSs. This limitation is particularly pronounced in tasks sensitive to the spatial distribution of lens flare and the specific characteristics of the affected objects.

To address these limitations, we propose a reference-free framework that leverages deep learning to estimate the “impact of lens flare”. This approach accurately captures visual degradation and its consequent effect on detector performance in the absence of reference images. Ultimately, we aim to enhance object detection in real-world autonomous driving scenarios by integrating our proposed module into existing detection frameworks, thereby improving robustness against lens-flare-induced artifacts.

**Figure 2:**
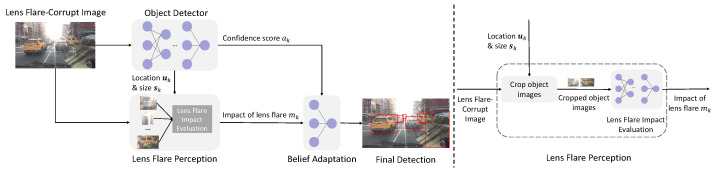
Pipeline of the proposed reference-free framework. This approach is designed to mitigate lens flare interference in object detection and is compatible with various detector architectures, including YOLO, Faster R-CNN, and DETR. The Lens Flare Perception Module first extracts regions of interest from the lens-flare-corrupted image based on the bounding box coordinates and dimensions provided by the base detector. It then quantifies the “impact of lens flare” as a scalar value. Subsequently, the Belief Adaptation Module leverages this scalar to recalibrate the original confidence scores, yielding the final detection results.

Figure 2 illustrates our proposed framework. In this work, we develop a reference-free method to enhance the performance of existing object detectors in the presence of lens flare. By leveraging information inherent in flare artifacts, our model strengthens analysis capabilities, effectively mitigating optical distortions and providing more accurate estimates of the “impact of lens flare”.

However, directly training a reference-free perception model to estimate the “impact of lens flare” is challenging. To circumvent this, we first develop a reference-based model—utilizing aligned pairs of lens-flare-free and lens-flare-corrupted images—to quantify this impact and optimize detector performance in an end-to-end manner. This reference-based model then serves as a teacher to supervise the training of the reference-free student model.

Our novelties are as follows:

- We propose a lightweight, reference-free model to estimate the “impact of lens flare” on detected objects, providing an efficient solution suitable for real-world applications;
- To develop the reference-free model, we employ a teacher–student training framework in which a reference-based teacher network guides the student model’s learning process;
- Our proposed method is detector-agnostic and can be seamlessly integrated into existing object detection frameworks to enhance performance in flare-affected scenarios with minimal training overhead;
- Unlike previous work relying on non-learnable pixel-level metrics [4], we introduce an end-to-end approach using a lightweight Convolutional Neural Network (CNN) to quantify the “impact of lens flare” and a three-layer Multi-Layer Perceptron (MLP) to optimize the detection via a Log-Likelihood Ratio (LLR) loss.

## 2. Background

### 2.1. Lens Flare and Related Work

**Figure 3:**
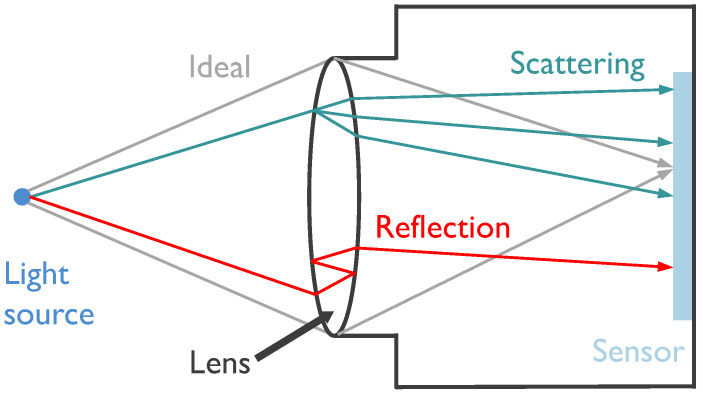
Light from a point source propagates along three distinct paths. A sharp image is formed on the sensor only when light follows the ideal optical path. Deviations from this path cause light to strike unintended regions of the sensor, resulting in lens flare artifacts. These artifacts typically manifest in two patterns: scattering and reflection. For instance, scratches or dust on the lens disrupt the uniformity of the refractive index, leading to scattered light. Furthermore, internal reflections at air-glass interfaces create additional parasitic paths, contributing to reflective flare.

Lens flare is an optical phenomenon occurring within imaging systems, characterized by the scattering or reflection of intense light rays [3]. These rays deviate from their intended optical paths and impinge upon the sensor at unintended locations, resulting in the manifestation of lens flare. Figure 3 illustrates the formation of lens flare within a typical optical system. In the context of autonomous driving, operational environments are often harsh; for instance, camera lenses frequently accumulate dust or grime. Consequently, lens flare is likely to occur in the presence of strong light sources, particularly in aging optical systems that have been in service for extended periods.

Extensive research has been conducted in the field of lens flare [4,5,6,7,8,9,10], with a primary focus on lens flare removal. Early studies predominantly relied on non-data-driven techniques until the introduction of the dataset by Wu et al. [5]. Subsequently, the availability of more comprehensive datasets [7,8] facilitated the development of advanced data-driven approaches. These advancements have improved the mitigation of complex lens flares consisting of diverse optical artifacts without necessitating additional hardware, as demonstrated in recent studies [11,12,13]. Although data-driven image preprocessing (i.e., de-flaring) models have achieved notable performance gains in visual restoration, they are typically architecturally complex and computationally intensive, resulting in high inference latency that hinders their deployment in real-time autonomous driving. More critically, as demonstrated in Section 5.2, such methods often suffer from information loss, where essential luminous objects like traffic lights are erroneously removed or distorted during the “de-flaring” process. This “black-box” approach to restoration without characterizing the underlying uncertainty can lead to crucial perception failures in safety-critical scenarios.

However, limited research has addressed the specific impact of lens flare on object detection. While our previous study [4] was among the first to mitigate these effects, its performance gains were constrained by a reliance on flare-free reference images, rendering it impractical for real-world deployment. In contrast, the current work achieves superior performance utilizing a reference-free, lightweight model that imposes minimal computational overhead.

### 2.2. Object Detection in Adverse Conditions

In autonomous driving, operational environments are highly complex, where factors such as varying weather, lighting conditions, dust, and lens scratches pose significant challenges to object detection. As a fundamental task in computer vision and autonomous driving, object detection has seen substantial improvements over time, driven by its critical importance in ensuring reliable performance under diverse conditions [14].

Since the introduction of the first CNN-based object detector, the Region-based Convolutional Network (R-CNN) [15], general object detection models have advanced significantly across various datasets. These developments encompass two-stage detectors such as R-CNN [15], Fast R-CNN [16], and Faster R-CNN [17]; one-stage detectors like You Only Look Once (YOLO) [18,19,20,21]; and the latest Transformer-based detectors such as the DEtection TRansformer (DETR) [22], followed by various improved versions of Transformer-based models [23,24,25,26].

However, these models are typically optimized for standardized benchmarks, such as the ImageNet [27], COCO [28], Waymo [29], and KITTI [30] datasets. Consequently, their robustness can be compromised when encountering corner cases—such as lens flare and overexposure—that are often overlooked by general-purpose object detectors. In autonomous driving, the robustness of these detectors is critical due to the inherent complexity of real-world environments. Therefore, extensive research has shifted toward targeted optimizations of general detectors to enhance their reliability across diverse and adverse conditions.

Adverse conditions, such as rain [31], fog or haze [32], snow [33], overexposure [34], and low lighting [35], are among the most extensively studied challenges in object detection. Most researchers concentrate on addressing a single type of issue, developing specialized models tailored to specific conditions. Furthermore, Chen et al. [36] proposed a unified model capable of handling multiple adverse weather conditions simultaneously, aiming to provide a more comprehensive and versatile solution for dynamic environments.

Although extensive research has addressed corner cases in object detection, studies specifically focused on lens flare remain scarce. However, lens flare is a non-negligible issue, particularly for vehicles with long service lives where lenses have accumulated scratches or dust. Such severe lens flare can significantly impact object detection and downstream decision-making.

## 3. Methodology

We propose an algorithm designed to enhance the robustness of generic object detectors against lens flare. The framework incorporates a reference-free lens flare perception model—utilizing only the input images (whether flare-free or flare-corrupted)—to estimate the impact of lens flare on each candidate detection. This estimate is subsequently processed by a belief adaptation module, which adjusts the detection outcomes to account for the perceived flare effects, thereby yielding flare-compensated results.

To develop the reference-free approach, we first require a reference-based model. As established in Section 1, this model leverages comparisons between original flare-free images and their flare-augmented counterparts.

Consequently, the proposed algorithm comprises three primary components: a reference-based lens flare perception model, a reference-free counterpart, and their respective LLR prediction models. The first two components serve as alternative implementations of the Lens Flare Perception module illustrated in Figure 2, both of which are designed to quantify the “impact of lens flare”.

Given a dataset containing images that may exhibit lens flares of varying shapes, locations, and intensities, a baseline object detector generates initial predictions—comprising object classes, bounding boxes, and confidence scores—which reflect the likelihood of a detection corresponding to an actual road user. Our objective is to characterize the distribution of positive and negative proposals across different levels of flare intensity. This is facilitated by a “lens flare perception” model, which guides an adaptation process to refine detection scores and enhance the reliability of object detection under challenging visual conditions.

Building on the Bayesian foundation of our previous work [4], this work presents three key innovations. First, we propose a reference-free framework that can remove the requirement for paired data. Second, we introduce a CNN-based metric to capture the specific impact of lens flares on object detection. Third, we integrate a novel loss function [37] for autonomous confidence calibration; this loss function is what enables the CNN-based metric to be trained in an end-to-end manner. Together, these advancements transition our approach from pixel-level restoration to a robust, detection-oriented solution for real-world driving.

### 3.1. Theoretical Foundation

We define a road user (e.g., pedestrians, cars, and trucks) using a tuple r=(x,g), where x denotes its position in the world coordinate system and g represents a feature vector providing additional information (such as color and size) for the road user.

We define an observation as $z_{k}=\left(u_{k},s_{k},a_{k}\right)$, where k∈{0,1,…,N} and *N* denotes the total number of observations. Here, $u_{k}$, $s_{k}$, and $a_{k}\in \left[0,1\right]$ represent the location, size, and confidence score of the *k*-th observation within a sensor-specific coordinate system, respectively. In the case of image-based object detectors, $u_{k}$ and $s_{k}$ are 2-element tuples specifying spatial coordinates and dimensions (i.e., width and height).

To account for lens flare, we define $m_{k}\in R$ to represent the “impact of lens flare” associated with the *k*-th observation. Since standard object detectors do not typically output $m_{k}$, we propose a trainable metric—computed via a lightweight CNN—to estimate $m_{k}$ for each detection. This enables a more precise quantification of the “impact of lens flare”. Further details of this approach are provided in the following subsection.

Two hypotheses are formulated to determine the presence or absence of a road user. For each observation $z_{k}$, the first hypothesis, $H_{1}\left(r,z_{k}\right)$, holds if a road user r exists with features similar to those of $z_{k}$ (focusing solely on size $s_{k}$, as the object detector only outputs size information for a given class), and if the projected image coordinates $\hat{u}$ of the real-world location x of r are close to those of $z_{k}$. Conversely, if no corresponding road user r exists for an observation $z_{k}$—implying that $H_{1}\left(r,z_{k}\right)$ does not hold—we define this via the null hypothesis $H_{0}\left(r,z_{k}\right)$. For brevity, these are hereafter referred to as $H_{1}$ and $H_{0}$.

To simplify modeling and computation, we assume that the location $u_{k}$ and size $s_{k}$ are independent of $a_{k}$ and $m_{k}$. We compute the posterior probabilities $P_{H|A,M}\left(H|a_{k},m_{k}\right)$ for $H_{1}$ and $H_{0}$, respectively, to determine the presence or absence of a road user r corresponding to an observation $z_{k}$. Based on Bayesian Theory, it can be reformulated as

(1) $$\frac{P_{A,M|H}\left(a_{k},m_{k}|H_{1}\right)}{P_{A,M|H}\left(a_{k},m_{k}|H_{0}\right)}>\frac{P(H_{0})}{P(H_{1})}.$$

If this inequality holds, $H_{1}$ is accepted; otherwise, $H_{0}$ is selected. The left-hand side of Equation (1) represents the likelihood ratio between the two hypotheses, while the right-hand side denotes the prior ratio, which is assumed to be a constant.

Similar to most research, we introduce log to Equation (1) to ensure the numeric stability. The resulting left-hand side becomes the LLR.

For each pair $\left(a_{k},m_{k}\right)$, the corresponding LLR value replaces $a_{k}$ to more accurately reflect the actual distribution of positive and negative detection proposals. This substitution accounts for variations in both the intensity and spatial location of the lens flare relative to the candidate detection.

### 3.2. “Impact of Lens Flare” Estimation

First, we describe how the “impact of lens flare” $m_{k}$ is derived using the reference-based approach, which utilizes both flare-corrupted and flare-free images. We assume that a flare-free image, $I_{\mathrm{clean}}$, and its flare-corrupted counterpart, $I_{\mathrm{flare}}$, are available for the same scenario and are spatially aligned (e.g., through simulation, as detailed later in this paper). For each detection output from $I_{\mathrm{flare}}$, the corresponding bounding box (BB) regions are cropped from both images:

(2) $$\begin{array}{c} {\mathrm{BB}}_{\mathrm{clean},k}=\mathrm{Crop}\left(I_{\mathrm{clean}},z_{k}\right),\\ {\mathrm{BB}}_{\mathrm{flare},k}=\mathrm{Crop}\left(I_{\mathrm{flare}},z_{k}\right),\\ k\in \{0,1,\ldots ,N\}. \end{array}$$

By employing a shared, lightweight feature extraction network, ${\mathrm{CNN}}_{\mathrm{extract}}$, we obtain per-layer feature activations: $F_{\mathrm{clean},k}$ for the flare-free bounding box ${\mathrm{BB}}_{\mathrm{clean},k}$, and $F_{\mathrm{flare},k}$ for the flare-corrupted bounding box ${\mathrm{BB}}_{\mathrm{flare},k}$:

(3) $$\begin{array}{c} F_{\mathrm{clean},k}={\mathrm{CNN}}_{\mathrm{extract}}\left({\mathrm{BB}}_{\mathrm{clean},k}\right),\\ F_{\mathrm{flare},k}={\mathrm{CNN}}_{\mathrm{extract}}\left({\mathrm{BB}}_{\mathrm{flare},k}\right). \end{array}$$

The difference between $F_{\mathrm{clean},k}$ and $F_{\mathrm{flare},k}$ is calculated for each layer as follows:

(4) $$\Delta F_{k,l}={\mathrm{CNN}}_{\mathrm{diff},l}\left(|F_{\mathrm{clean},k,l}-F_{\mathrm{flare},k,l}|\right),l\in \left\{1,\ldots ,L\right\},$$

where |·| denotes an element-wise absolute difference.

By processing $\Delta F_{k,l}$ through pooling layers and/or non-linear activation functions, the feature representations are projected onto a single scalar value. This process yields the corresponding $m_{k}$ for the *k*-th detector output, effectively encapsulating the lens flare impact.

This enables $m_{k}$ to only encapsulate the variations induced by lens flare, serving as a quantifiable measure of its impact on detection performance. The CNN architecture is optimized to only capture the subtle changes caused by lens flare, making $m_{k}$ a reliable indicator of its effect.

**Figure 4:**
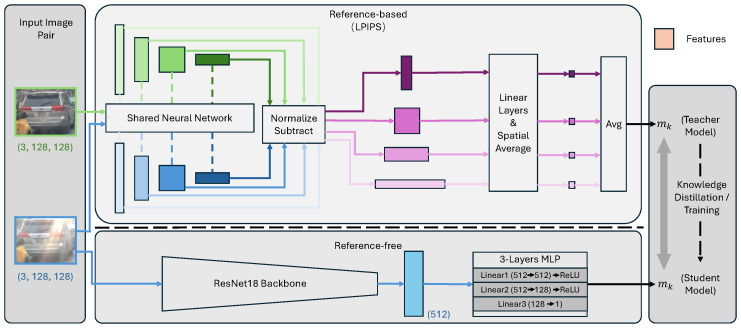
Overview of the “impact of lens flare” $m_{k}$ estimation framework. The reference-based teacher model (**top**) employs an LPIPS-based architecture [38] to extract features from image pairs. The reference-free student model (**bottom**), consisting of a ResNet18 backbone [39] and a 3-layer MLP, is trained to estimate $m_{k}$ via knowledge distillation using the teacher’s output as supervision.

Equations (2)–(4) characterize the reference-based approach. In contrast, the reference-free method operates solely on flare-related features by directly inputting $F_{\mathrm{flare},k}$ from Equation (3) into an MLP to compute $m_{k}$. To ensure that the reference-free model exclusively leverages lens flare information, it is trained within a teacher–student framework, where the reference-based method serves as the teacher. Figure 4 provides a more intuitive illustration. Further training details are provided in Section 4.

### 3.3. Loss Function

#### 3.3.1. LLR Loss

In practical applications, obtaining the exact analytical forms of the likelihood functions for the two hypotheses, $H_{0}$ and $H_{1}$, is often challenging. However, assuming the availability of a training dataset that sufficiently reflects real-world distributions (see Section 4 for a detailed discussion), we can adopt a data-driven approach to address this problem. While this could be implemented via histogram-based methods or Kernel Density Estimation (KDE), we employ an MLP to achieve a more precise estimation of the LLR. This choice is motivated by the fact that MLPs are less sensitive to hyperparameter selection (e.g., bandwidth in KDE), a conclusion supported by the ablation study in Section 5.3. Furthermore, the use of an MLP facilitates end-to-end training, which is essential for obtaining a trainable metric to estimate $m_{k}$.

Consequently, we employ the loss function derived in [37] to train our model in an end-to-end manner. Given that the complete derivation in [37] is quite intricate, we provide a concise summary in this section for clarity. For a more comprehensive derivation, we refer the reader to the original work [37].

To adapt the general formulations from [37] to our proposed method and streamline the notation, we first introduce the following definitions:

(5) $$\begin{array}{cc} f_{0}\left(X\right) & =p(a_{k},m_{k}|H_{0})\\ f_{1}\left(X\right) & =p(a_{k},m_{k}|H_{1}), \end{array}$$

(6) $$X=(a_{k},m_{k}),$$

where $f_{0}\left(X\right)$ and $f_{1}\left(X\right)$ denote the likelihood functions corresponding to hypotheses $H_{0}$ and $H_{1}$, respectively, defined over the 2-dimensional vector $X=(a_{k},m_{k})$.

As established in [37], if the loss function is formulated as

(7) $$L_{\mathrm{llr}}\left(X\right)=\frac{1}{N_{0}}\sum_{i=1}^{N_{0}}e^{-0.5\mathrm{MLP}\left(X_{i}^{0}\right)}+\frac{1}{N_{1}}\sum_{j=1}^{N_{1}}e^{0.5\mathrm{MLP}(X_{j}^{1})},$$

where $X_{i}^{0}$ and $X_{j}^{1}$ denote samples drawn from $f_{0}\left(X\right)$ and $f_{1}\left(X\right)$, respectively; $N_{0}$ and $N_{1}$ represent the number of samples associated with $H_{0}$ and $H_{1}$; and MLP(X) is a MLP taking X as input. We can approximate the desired LLR function of the two likelihood functions shown as

(8) $$\mathrm{MLP}\left(X\right)\approx \log\frac{f_{0}\left(X\right)}{f_{1}\left(X\right)}.$$

Based on the definitions in Equation (5), this expression represents the LLR of the two hypotheses, $H_{1}$ and $H_{0}$, as formulated in Equation (1).

Ideally, the LLR distribution would be approximated using all available data in a single batch; however, this is infeasible due to hardware constraints. Instead, we implement the training in batches. Consequently, in the final formulation of the loss function, $N_{0}$ and $N_{1}$ are replaced by $n_{0}$ and $n_{1}$, where $n_{0}$ and $n_{1}$ denote the number of samples per batch for $H_{0}$ and $H_{1}$, respectively. To maintain consistency, we ensure that the condition $n_{0}/n_{1}=N_{0}/N_{1}$ is satisfied.

#### 3.3.2. Cross-Model Loss

Having initially trained the reference-based lens flare perception model, we leverage it to derive a reference-free counterpart via a teacher–student learning framework, which necessitates a cross-model loss. Since the outputs of both models are single scalar values, we employ the Smooth L1 loss for optimization as follows:

(9) $$L_{\mathrm{CM}}\left(\hat{m_{k}},m_{k}\right)=\left\{\begin{array}{cc} 0.5{\left(\hat{m_{k}}-m_{k}\right)}^{2} & \mathrm{if}\;|\hat{m_{k}}-m_{k}|<1\\ |\hat{m_{k}}-m_{k}|-0.5 & \mathrm{otherwise}, \end{array}\right.$$

where $\hat{m_{k}}$ and $m_{k}$ are the outputs from the reference-free lens flare perception model and the reference-based lens flare perception model, respectively.

## 4. Experimental Setup and Implementation

We aim to demonstrate that our approach achieves higher Average Precision (AP) with marginal computational overhead. Furthermore, we evaluate the performance of the reference-free lens flare belief adaptation system relative to the upper bound established by an oracle (reference-based) system.

### 4.1. Datasets

Due to the scarcity of large-scale autonomous driving benchmarks that provide paired flare-corrupted and ground-truth flare-free images, we follow the well-established data synthesis pipelines from state-of-the-art lens flare research [5,8,9,12] to develop our high-fidelity synthetic dataset. This approach ensures that our data generation method remains consistent with current research standards while allowing for a systematic and controllable evaluation of object detection robustness under diverse optical interference. Specifically, our pipeline integrates a foundational autonomous driving dataset with lens flare datasets, where isolated flare patterns are physically overlaid onto clean driving scenes to simulate realistic sensor degradation.

#### 4.1.1. Lens Flare Datasets

Optical characteristics of lens flares vary significantly depending on the illuminant conditions. During the day, flares are typically dominated by a single, high-intensity source (the sun). In contrast, nighttime scenarios involve multiple heterogeneous light sources, such as traffic lights, streetlamps, and vehicle headlights, which produce complex multi-colored flare patterns.

To replicate these effects, we utilize the Flare7K++ [8] dataset for nighttime simulations, which provides diverse patterns including glares, shimmers, and streaks. For daytime scenarios, we follow the methodology in [5] to generate a custom library that avoids the truncated light source issues found in earlier versions. These datasets have been widely adopted in flare removal studies [8,9,12], underscoring their effectiveness and physical realism. Crucially, while primarily synthetic, both daytime and nighttime sets incorporate authentic, real-world captured lens flares to bridge the domain gap and validate the practical applicability of our trained models.

#### 4.1.2. Autonomous Driving Datasets

We employ the BDD100K [40] dataset as our foundational benchmark for object detection. We intentionally exclude multi-sensor datasets like nuScenes [41] to maintain strict visual consistency; in such datasets, objects labeled via LiDAR or Radar may not be visually discernible in camera frames, which could introduce spurious errors unrelated to lens flare interference.

BDD100K provides the necessary environmental diversity and scale required for this task, encompassing a wide spectrum of driving trajectories and weather conditions. The dataset consists of high-resolution (720p) video frames and evaluates ten primary object classes: person, rider, car, truck, bus, train, motorcycle, bicycle, traffic light, and traffic sign. Its comprehensive nature ensures that our evaluation covers a representative range of real-world driving scenarios.

### 4.2. Implementation Details

**Figure 5:**
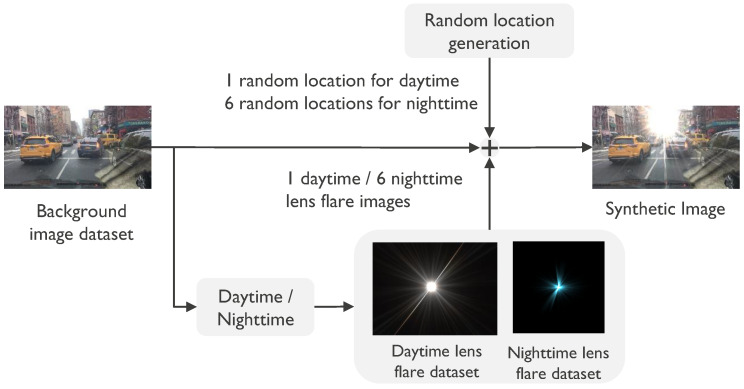
Pipeline for generating the lens-flare-corrupted dataset. To account for the distinct characteristics of light at different times, separate datasets are utilized for daytime and nighttime flares. Images are categorized by a pixel-intensity threshold. We constrain the number of added flare instances—limiting daytime images to one and nighttime to six—to reflect the typically smaller size and higher frequency of flares in nocturnal scenes. Flare coordinates are randomized, consistent with established methodologies in lens flare research.

**Figure 6:**
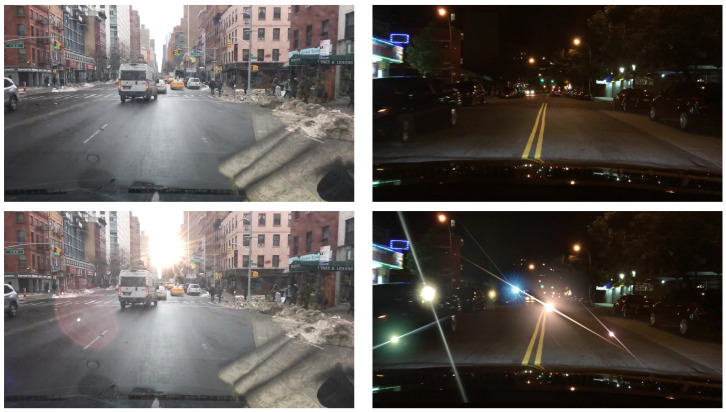
Synthetic lens flare examples for daytime and nighttime scenarios. The top row displays the original background images, while the bottom row shows the corresponding synthetic outputs. For daytime scenes, a single white lens flare is integrated; for nighttime scenes, six colorful flares are added to simulate multiple light sources.

The BDD100K dataset [40] comprises $7\times 10^{4}$ training, $1\times 10^{4}$ validation, and $2\times 10^{4}$ testing images. However, as the testing set does not provide publicly available ground-truth labels, it was excluded from our study. We utilize the medium-sized YOLOv5 model [19] as our baseline object detector due to its widespread adoption in both academia and industry. Throughout this paper, this baseline is referred to as the “original object detector”, which was trained on the BDD100K training set without lens flare augmentation. Our proposed method is evaluated using the BDD100K validation set, which is augmented with synthetic lens flare and partitioned into two subsets: one for training our proposed models and the other for testing. For each image in the validation set, we apply the pipeline illustrated in Figure 5 to generate flare-corrupted images. Specifically, for a given image, we first determine whether it depicts a daytime or nighttime scenario by counting pixels that exceed a predefined brightness threshold, and then select the corresponding lens flare from the dataset. We constrain the maximum number of added lens flares to one for daytime and six for nighttime, as nighttime flares are generally smaller. To create an expanded dataset, each image is processed six times with variations in the appearance and location of the lens flares. Figure 6 illustrates examples of these synthetic images for both daytime and nighttime scenarios.

We perform 5-fold cross-validation to evaluate the robustness of our approach. The Intersection over Union (IoU) threshold for positive detections is established at 0.5, while the confidence score threshold for the original object detector is set to $1\times 10^{-4}$ to ensure comprehensive candidate retrieval.

#### 4.2.1. Estimation of “Impact of Lens Flare” (Reference-Based)

**Figure 7:**
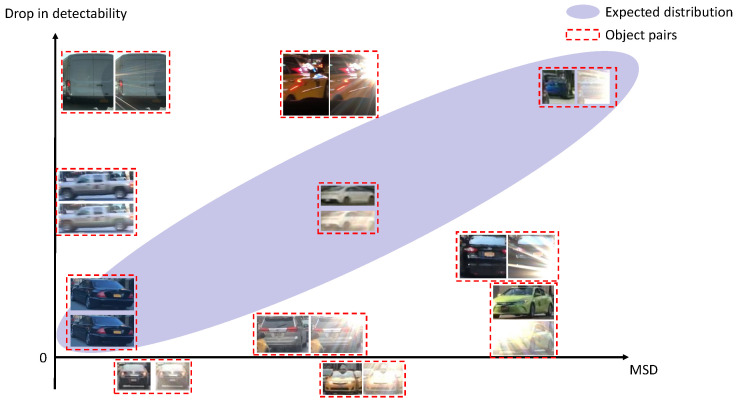
Comparison between a pixel-level metric (between perfectly aligned lens-flare-free and lens-flare-corrupted images) and the “drop in detectability” (reflected by the object detector’s confidence score). Red-dashed rectangles highlight representative outliers from the real-world dataset. These examples demonstrate that pixel-level discrepancies between perfectly aligned flare-free and flare-corrupted images fail to fully capture the actual impact of lens flare on object detection performance.

As demonstrated in Section 1 and Figure 7, pixel-level metrics such as MSD, Peak Signal-to-Noise Ratio (PSNR), and Structural Similarity (SSIM) between flare-free and flare-corrupted images fail to fully capture the impact of lens flare on object detection. This insufficiency stems from the inherent complexity of lens flare and the specific nature of the object detection task itself. Instead, we employ the Learned Perceptual Image Patch Similarity (LPIPS) metric [38] as a trainable estimator for $m_{k}$ for each detection output. The architecture of the LPIPS network is designed to evaluate the perceptual discrepancy between two input images, enabling the model to isolate information specifically introduced by the lens flare. Our observations indicate that the severity of the lens flare’s impact is multifaceted, depending on factors such as the spatial relationship between the flare and the object, as well as object-specific conditions (e.g., occlusion, truncation, or blurring). By computing differences at the feature level rather than the pixel level, LPIPS effectively captures these semantic nuances, providing a more robust quantification of the lens flare impact.

We fine-tuned the pretrained LPIPS network using the LLR loss function (Equation (7)) to enhance the extraction of discriminative features under lens flare conditions. By minimizing the LLR loss, the network learns to output consistent $m_{k}$ values when the distributions of $H_{1}$ and $H_{0}$ are similar, effectively focusing the model on the most informative features for hypothesis discrimination.

#### 4.2.2. Estimation of “Impact of Lens Flare” (Reference-Free)

**Figure 8:**
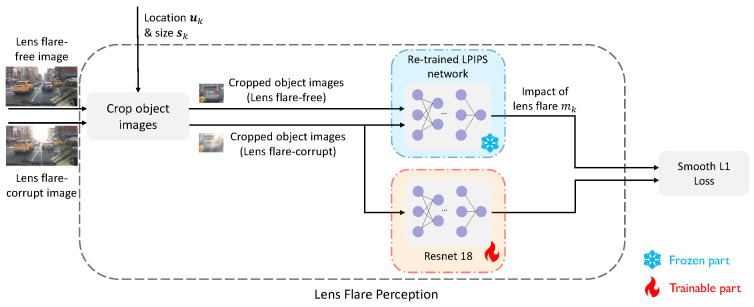
Training pipeline for the reference-free lens flare perception model. Building upon the reference-based model detailed in Section 4.2.1, we propose a reference-free alternative guided by a teacher–student framework. To eliminate the requirement for flare-free reference images, the LPIPS-based teacher is replaced by a ResNet18-based perception module (the student), which operates solely on flare-corrupted inputs. The training is optimized using the Smooth L1 loss, as defined in Equation (9).

In realistic autonomous driving scenarios, it is impossible to simultaneously obtain flare-free and flare-corrupted images for the same instance. Consequently, the development of a reference-free model is essential. Leveraging the reference-based model derived in Section 4.2.1, we employ a teacher–student framework to train a single-image input network, as illustrated in Figure 8. In this configuration, the fine-tuned LPIPS network serves as the teacher, while the reference-free model acts as the student. Specifically, we utilize ResNet18 [39] as the backbone, followed by a three-layer MLP to predict the scalar $m_{k}$ from the extracted features. As shown in Figure 2, this ResNet18-based module functions as the “Lens Flare Perception” component.

#### 4.2.3. Prediction of LLR

Following the Bayesian framework established in Section 3.1, we refine the candidates from the baseline object detector by computing the LLR. This calculation is based on the confidence score $a_{k}$, provided by the original detector, and the perception metric $m_{k}$, which is derived using the reference-based or reference-free methods detailed in Section 4.2.1 and Section 4.2.2, respectively.

This task is essentially a probability density estimation problem in a two-dimensional space. Given its manageable complexity, the LLR can be effectively modeled using a compact architecture; thus, we employ a three-layer MLP for this purpose. Specifically, the MLP architecture consists of an input layer accepting the $\left(a_{k},m_{k}\right)$, followed by two hidden layers each containing 20 neurons, and a final output layer with a single neuron (2→20→20→1). LeakyReLU activation functions are utilized between the layers to introduce non-linearity while mitigating the vanishing gradient problem. Utilizing an MLP facilitates a smoother and more precise LLR estimation across the continuous input domain. Crucially, the differentiability of the MLP ensures that the gradient of the LLR loss (Equation (7), derived in Section 3.3) can be backpropagated to the LPIPS network, enabling end-to-end optimization of the entire perception framework.

### 4.3. Training Pipeline

The comprehensive training pipeline of this work is summarized in Algorithm 1. Utilizing the input dataset, we first develop a reference-based lens flare perception model, which subsequently serves as the teacher within a teacher–student framework to facilitate the training of a reference-free counterpart. This structured transition ensures the effective transfer of knowledge from reference-dependent to reference-free learning. The specific training strategies for both models are detailed in the following sections.

To develop the reference-based model, we initiate a joint training phase for the LPIPS network and the MLP over several initial epochs. Subsequently, the LPIPS network parameters are frozen, and the MLP alone is trained for an additional several thousand epochs to refine the LLR estimation. Given the compact architecture of the MLP, training for $1\times 10^{4}$ epochs remains computationally efficient, typically concluding within tens of minutes. The specific training duration is tailored to each object class to ensure loss convergence. For instance, for the “car” class, the LPIPS network is optimized for 2 epochs, followed by $1\times 10^{4}$ epochs of MLP training. Notably, a dedicated LPIPS network and an MLP are trained for each distinct class.

As illustrated in Figure 8, the reference-free lens flare perception model is trained via a teacher–student paradigm using the cross-model loss (Equation (9)) on the same data split. Subsequently, we integrate the trained reference-free model with an MLP. By freezing the parameters of the perception model, we train the MLP to predict the LLR, mirroring the training procedure of the reference-based model. The key distinction, however, is that this stage operates solely on single flare-corrupted images. We continue to optimize the MLP for several thousand epochs, with the specific duration tailored to each object class to ensure convergence. For the “car” class, the reference-free perception model is trained for 3 epochs, followed by $1\times 10^{4}$ epochs for the MLP.
**Algorithm 1:** Training Pipeline**Initialization:**Freeze detector parameters.Initialize the Reference-Based lens flare perception model (RB)with pre-trained parameters from [38].Randomly initialize MLP_RB_.Set X←[].**Reference-Based Training:****for** *epoch = 1* **to** *$N_{\mathrm{RB}}$* **do**      **foreach** $(I_{\mathrm{clean}},I_{\mathrm{flare}})\in D$ **do**             $\left(BB,a_{k}\right)\leftarrow \mathrm{Detector}\left(I_{flare}\right)$;             $m_{k\_\mathrm{RB}}\leftarrow \mathrm{RB}\left(I_{\mathrm{clean}},I_{\mathrm{flare}},BB\right)$;             $L_{\mathrm{llr}}\left({\mathrm{MLP}}_{\mathrm{RB}}\left(a_{k},m_{k\_\mathrm{RB}}\right)\right)$;             Update parameters;      **end****end**Load best RB weights and freeze RB;**foreach** $(I_{\mathrm{clean}},I_{\mathrm{flare}})\in D$ **do**      $\left(BB,a_{k}\right)\leftarrow \mathrm{Detector}\left(I_{\mathrm{flare}}\right)$;      $m_{k\_\mathrm{RB}}\leftarrow \mathrm{RB}\left(I_{\mathrm{clean}},I_{\mathrm{flare}},BB\right)$;      Append $\left[a_{k},m_{k\_\mathrm{RB}}\right]$ to *X*;**end****for** *epoch = 1* **to** *$N_{\mathrm{MLP}}$* **do**      $L_{\mathrm{llr}}\left({\mathrm{MLP}}_{\mathrm{RB}}\left(X\right)\right)$;      Update MLP_RB_;**end****Reference-Free Training:**Initialize the Reference-Free lens flare perception model (RF)with parameters from [39].Randomly initialize MLP_RF_.Reset X←[].**for** *epoch = 1* **to** *$N_{\mathrm{RF}}$* **do**      **foreach** $(I_{\mathrm{clean}},I_{\mathrm{flare}})\in D$ **do**             $\left(BB,a_{k}\right)\leftarrow \mathrm{Detector}\left(I_{\mathrm{flare}}\right)$;             $m_{k\_\mathrm{RB}}\leftarrow \mathrm{RB}\left(I_{\mathrm{clean}},I_{\mathrm{flare}},BB\right)$;             $m_{k\_\mathrm{RF}}\leftarrow \mathrm{RF}\left(I_{\mathrm{flare}},BB\right)$;             $L_{\mathrm{CM}}\left(m_{k\_RB},m_{k\_\mathrm{RF}}\right)$;             Update RF using $L_{\mathrm{CM}}$;      **end****end**Load best RF weights and freeze RF;**foreach** $I_{\mathrm{flare}}\in D$ **do**      $\left(BB,a_{k}\right)\leftarrow \mathrm{Detector}\left(I_{\mathrm{flare}}\right)$;      $m_{k\_\mathrm{RF}}\leftarrow \mathrm{RF}\left(I_{\mathrm{flare}},BB\right)$;      Append $\left[a_{k},m_{k\_RF}\right]$ to *X*;**end****for** *epoch = 1* **to** *$N_{\mathrm{MLP}}$* **do**      $L_{\mathrm{llr}}\left({\mathrm{MLP}}_{\mathrm{RF}}\left(X\right)\right)$;      Update MLP_RF_;**end**

## 5. Result and Discussion

We demonstrate that the proposed method significantly enhances the AP of the baseline object detector in flare-affected scenes. A common alternative for improving detection performance in such contexts is to pair an off-the-shelf detector with a specialized lens flare removal algorithm. We evaluate our approach against this alternative and show that our solution is more hardware-efficient and offers superior time efficiency compared to incorporating a dedicated lens flare removal module.

### 5.1. Average Precision

**Figure 9:**
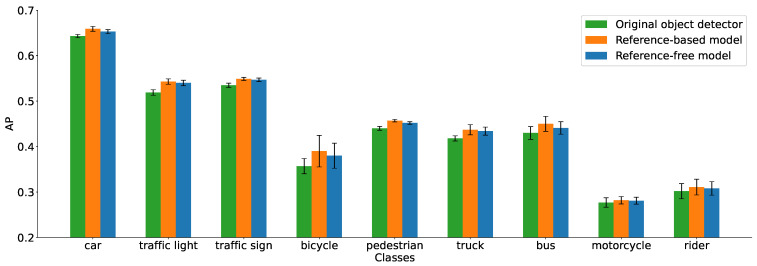
Comparative performance of various models across different categories, as detailed in Table 1. For enhanced visualization of the performance gaps, the AP axis begins at 0.2.

Table 1 presents the results for the original object detector alongside the enhanced versions incorporating our “lens flare perception” network, covering both reference-free and reference-based approaches. For a more intuitive comparison across various categories, these performance differences are visualized in the bar plot in Figure 9. We evaluated the AP for all classes in the synthetic BDD100K dataset augmented with lens flare, excluding the “train” class due to its insufficient number of detections. Across the 5-fold cross-validation and all evaluated classes, both the reference-based and reference-free models consistently outperform the original detector. While the reference-based model achieves superior performance, the reference-free model follows closely as the second best. Notably, for certain categories such as “traffic sign”, the reference-free model performs almost on par with its reference-based counterpart.

The performance gains across different classes vary primarily due to two factors. First, the impact of lens flare is inherently class-dependent. For instance, at night, lens flares may be misidentified as vehicles because headlights often appear as pairs of high-intensity light spots; similarly, traffic lights are themselves sources of lens flare. Second, the scarcity of training instances for certain categories in the dataset limits potential improvements. Nevertheless, even for underrepresented classes such as the “motorcycle” class, a marginal yet discernible improvement is still observed.

While our proposed method achieves consistent performance gains across all evaluated object categories (as detailed in Table 1), the following in-depth analysis focuses primarily on the “car” class. This choice is motivated by the inherent long-tail distribution of the BDD100K dataset, where certain classes (e.g., train, rider) contain insufficient instances for a reliable, detailed analysis. This “car” category, being the most frequent, provides a statistically significant and robust basis for investigating the complex interactions between lens flare and detector confidence. Consequently, the results for the “car” class serve as the strongest and most representative evidence for the effectiveness of our framework, while the generalizability is confirmed by the overall AP improvements in Table 1.

Compared with the original object detector, the reference-based model yields a 1.6% improvement in AP. While transitioning to the reference-free model results in a 0.6% decrease in AP, it still outperforms the original detector by a margin of 1.0%. This outcome is consistent with our expectations; when the reference-free model is trained alongside the reference-based model, it cannot fully encapsulate all the informative features that the reference-based counterpart derives from a single input.

**Figure 10:**
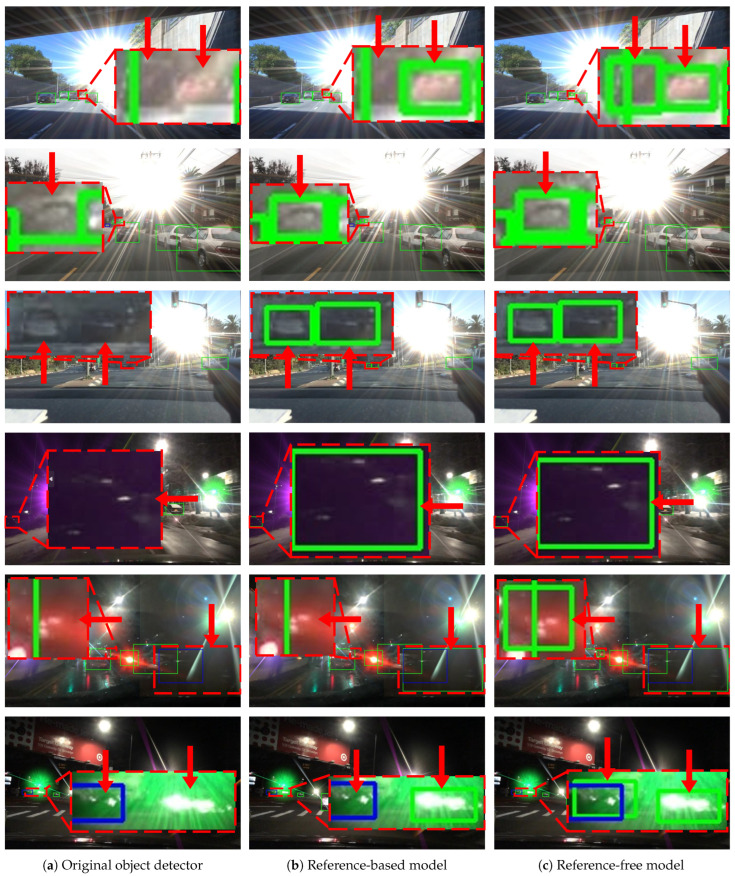
Detection examples for the “car” class. Green and blue rectangles denote TP and FP detections, respectively. These results reflect a realistic deployment scenario where a score threshold determines the final output. In this analysis, precision is fixed at 0.7 to ensure a consistent false positive rate across all models. Superior performance at a fixed precision is indicated by higher recall, manifested as an increase in TPs without additional FPs. Under both daytime and nighttime conditions, the proposed reference-free and reference-based models exhibit improved recall compared to the baseline detector.

Figure 10 illustrates representative detection results for the “car” class, contrasting the performance of the original detector with our reference-free and reference-based models under both daytime and nighttime conditions. In these visualizations, green and blue bounding boxes denote True Positive (TP) and False Positive (FP) detections, respectively.

**Figure 11:**
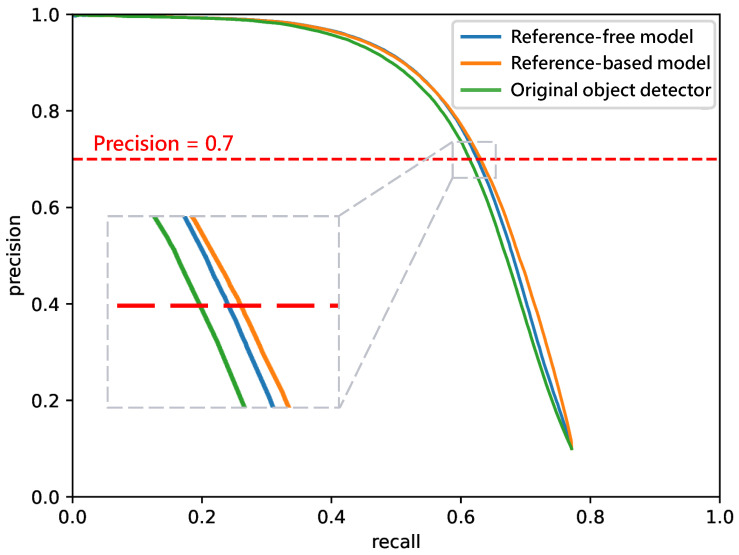
PR curves for the “car” class. This analysis simulates a realistic deployment scenario where a confidence threshold is applied to the detection score to make a definitive decision. By fixing the precision at 0.7, the corresponding score thresholds are determined for each model to identify object presence. At this precision level, the reference-based and reference-free models achieve recall gains of 0.017 and 0.012, respectively.

These results reflect a practical deployment scenario where a specific score threshold is applied to determine the final output. Specifically, the precision is fixed at 0.7 to derive the corresponding operating thresholds for the original, reference-free, and reference-based models. These thresholds are subsequently employed to generate the final detection decisions. The corresponding Precision-Recall (PR) curves are depicted in Figure 11. As illustrated, at a fixed precision of 0.7, the reference-based and reference-free models yield recall improvements of 0.017 and 0.012, respectively, compared to the original one.

In Figure 10, key regions are magnified and highlighted with red-dashed rectangles to emphasize specific details. These visual results demonstrate that both the reference-free and reference-based models successfully identify additional TPs without increasing the number of FPs under both daytime and nighttime conditions.

### 5.2. Time Efficiency

Our proposed method is compared against a baseline approach that incorporates lens flare removal as a preprocessing stage before object detection. The results of this comparison are summarized in Table 2 and Table 3. For the flare removal task, we employ a State-Of-The-Art (SOTA) transformer-based model as described in [8].

**Figure 12:**
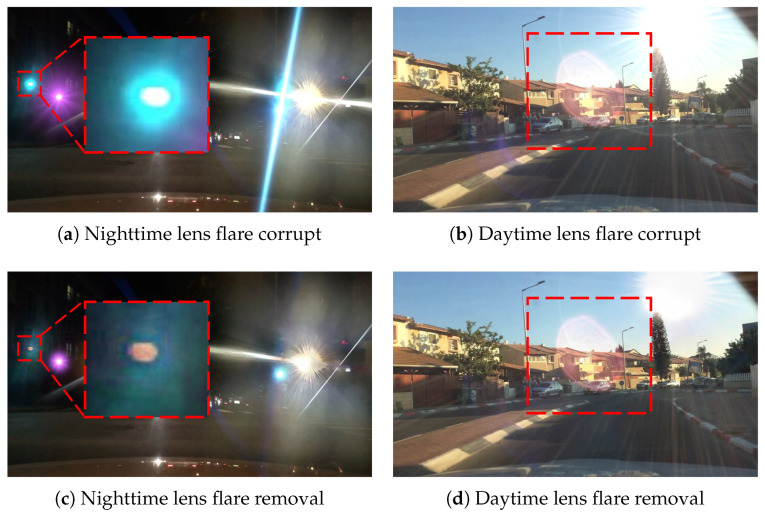
Representative failure cases of the lens flare removal model in [8], illustrating that lens flare removal alone may be insufficient under challenging conditions. Note that the proposed method does not perform lens flare removal; instead, it operates as a post-processing strategy on the detector outputs. In (**a**) and (**c**), the red dashed rectangles highlight traffic lights that become nearly invisible after flare removal. While (**b**) shows a realistic lens flare, (**d**) presents the processed result where the flare is still inadequately suppressed. A similar artifact can also be observed in the right-hand region of (**c**).

Table 2 presents the AP results for the “car” class, evaluated using YOLOv5m on both lens-flare-corrupted images and their de-flared counterparts across the BDD100K validation set. While the improvements achieved via lens flare removal appear to surpass those of our proposed models, these results should be interpreted as being artificially inflated. This is primarily because most lens flares in our experiments originate from the same public dataset used in that model’s training pipeline. Figure 12 presents representative failure cases of this SOTA lens flare removal method, showing that removal-based preprocessing alone may not sufficiently address detection degradation. Figure 12b,d illustrate failure cases where the model struggles with complex, real-world flare patterns. Furthermore, Figure 12a,c highlight a critical drawback: the removal model may erroneously eliminate traffic lights or distort their colors. This is a recognized issue for lens flare removal models [9] and poses substantial safety risks in autonomous driving scenarios.

Table 3 reports the per-image inference time of our proposed method compared with existing lens-flare removal approaches. Since our method operates as a lightweight post-processing module built upon the detector outputs, rather than performing full image restoration, it follows a fundamentally different computational paradigm from lens-flare removal networks. To more comprehensively demonstrate the efficiency advantages of our design, we evaluate not only the SOTA transformer-based restoration model discussed earlier but also an additional SOTA CNN-based lens-flare removal method [13], representing two typical architectural families in this task. To more accurately reflect a practical autonomous-driving pipeline, we measure only the core computational stages for each model. All peripheral overheads, such as frame I/O, data formatting, and tensorization, are excluded to ensure that the comparison focuses solely on intrinsic model efficiency.

Experimental analysis reveals that the primary computational bottleneck in our proposed method is the extraction of detection box patches from the full frame, a consequence of the dense detection proposals. In contrast, the LPIPS/ResNet-18 and MLP inference stages account for only approximately 10% of the total processing time. As indicated in Table 3, the relatively low GPU footprint of our models facilitates the parallel execution of multiple inference instances across different categories. Consequently, in this study, while patches were cropped for all classes, inference was performed exclusively for the “car” class, which represents the most prevalent category in our dataset. All experiments were conducted on a workstation equipped with an Intel i7-11700KF CPU and an NVIDIA RTX 3080 Ti GPU.

As illustrated in Table 3, our proposed post-processing stages, both reference-free and reference-based, are nearly 10× faster than the SOTA lens flare removal preprocessing approaches. The latter introduces a significant overhead of 191 ms and 201 ms per frame, resulting in a frame rate of approximately 5 FPS; such latency is prohibitively high for real-time autonomous driving applications. In contrast, our method requires only 19 ms to process a single frame and, owing to its minimal memory footprint, facilitates parallel execution across multiple categories.

### 5.3. Ablation Study

To validate the necessity of employing a learnable metric (LPIPS) over a pixel-level metric for estimating $m_{k}$ and a lightweight MLP with an LLR loss function instead of a histogram-based method for LLR prediction, we conducted four ablation experiments. The results are summarized in Table 4. Specifically, we evaluated the combinations of two metrics—perceptual similarity (LPIPS) and pixel-level difference (MSD)—with two LLR estimation techniques: histogram-based KDE and the MLP. This yielded four configurations: LPIPS+KDE, LPIPS + MLP, MSD + KDE, and MSD + MLP. Notably, when utilizing LPIPS with KDE, retraining the LPIPS model is infeasible because the loss function gradients cannot be backpropagated through the KDE component to the LPIPS network. Consequently, pre-trained LPIPS weights from [38] were adopted for this configuration.

As shown in Table 4, when employing MSD as the metric for $m_{k}$, the MLP-based LLR prediction outperforms the KDE-based approach by 0.002. Using the LPIPS metric yields AP improvements of 0.001 (with KDE) and 0.013 (with MLP) compared to the MSD-based baseline, highlighting the effectiveness of this learnable metric. Furthermore, the results indicate that the MLP consistently achieves superior AP performance compared to KDE, regardless of whether MSD or LPIPS is used for $m_{k}$ estimation. The performance gain is particularly significant for the LPIPS + MLP combination (+0.012), as it facilitates end-to-end retraining under the LLR loss. This enables LPIPS to learn more discriminative features specifically tailored for lens flare scenarios.

### 5.4. Generalization Across Diverse Detection Architectures

To further substantiate the effectiveness and robustness of our proposed framework, we extended our evaluation beyond YOLOv5m to include several recent SOTA detection models, specifically YOLOv10-medium [42], YOLOv11-medium [43], YOLOv12-medium [44], and the Transformer-based RT-DETR-large [45]. It is important to note that YOLOv12 represents a transformative shift in the series’ backbone by incorporating advanced attention mechanisms. This architectural evolution enables the network to capture long-range spatial dependencies in a manner similar to DETRs. Consequently, the inclusion of YOLOv12—alongside RT-DETR—ensures that our proposed method is rigorously validated against both strictly convolutional and attention-based hybrid frameworks.

In this experiment, the object detection models were initialized with weights pre-trained on the COCO dataset without fine-tuning. As different detectors exhibit distinct behavioral patterns and error distributions when subjected to flare corruption, we retrained the lens flare perception and LLR prediction modules for each specific detector. To maintain experimental consistency, the training protocol for all detectors followed the same procedure as the YOLOv5m, employing 5-fold cross-validation on the synthetic lens-flare-corrupted BDD100K validation set. We focused on the “car” class—a major overlapping category between the COCO and BDD100K datasets.

As summarized in Table 5, our reference-free and reference-based modules consistently yield performance gains across all tested architectures. Notably, when integrated with an SOTA detector such as YOLOv12, our method yields an AP improvement of 0.02 (reference-based) and 0.019 (reference-free). These results demonstrate that our lens-flare-aware approach is model-agnostic and exhibits robust generalization across different detector architectures. Consequently, it confirms that our framework can be seamlessly integrated as a plug-and-play enhancement for a wide range of modern object detectors in real-world autonomous driving scenarios.

### 5.5. Evaluation of Real-World Generalization

To validate the real-world applicability, we evaluated our framework on the original BDD100K training set. Although the original BDD100K images were not augmented with lens flares, they inherently contain a small proportion of natural, physical lens flare. It should be emphasized that this portion of the data was entirely excluded from the training phases of both our model (Section 5.4) and the baseline detectors. Consequently, the network had no prior exposure to these specific samples, ensuring an unbiased evaluation. We randomly select $1\times 10^{4}$ images to obtain the results.

Since we do not have paired flare-free and flare-corrupted images in this case, we leverage the reference-free weights obtained via Teacher–Student distillation on synthetic data. The weights for the reference-free module were directly adopted from the final fold of the 5-fold cross-validation described in Section 5.4, rather than selecting the optimal (best-performing) fold. This choice ensures that the results reflect the general stability of our approach rather than a cherry-picked peak performance.

Theoretically, since our method only adjusts confidence in identified interference regions and preserves original detections elsewhere, the resulting AP should be at least equal to or greater than the baseline, depending on the number of lens flares and the detector’s sensitivity to them. As shown in Table 6, our approach consistently improves performance across various SOTA architectures, including YOLOv12 and RT-DETR. This confirms the effectiveness of our framework on real-world datasets.

### 5.6. Limitation

Despite the consistent performance improvements across various SOTA detectors, it is essential to discuss the inherent limitations of the proposed framework. Our approach is designed as a post-processing optimization method that operates on the candidate proposals provided by a baseline detector. Consequently, it does not modify the internal feature extraction or the initial proposal generation stages.

Impact on False Negatives (FNs): The impact of our method on FNs is two-fold. Since a FN is defined as an object falling below the detection threshold, our framework can effectively recover FNs that were initially proposed by the detector head but assigned a low confidence score due to flare interference. By recalibrating these scores based on flare physics, our method brings such “missed” candidates back above the threshold. However, a limitation remains for “totally vanished” objects: if an object is so severely obscured that the detector head fails to generate any candidate proposal, our module has no input to optimize and thus cannot “restore” the detection. In these extreme cases, the limitation lies in the baseline detector’s inability to perceive the region’s latent features. Nonetheless, the proposed framework represents a best-effort utility of the existing camera and detector setup. By effectively calibrating the confidence scores of all available candidates, it ensures a higher overall AP and a more reliable output compared to a flare-ignorant system.

Synergy with Sensor Fusion: For objects entirely obscured by lens flare (the “totally vanished” cases), multi-modal fusion (e.g., LiDAR or Radar) is a more robust solution than relying purely on visual information. As shown in Figure 12, even SOTA Transformer-based flare removal [8] can erroneously remove critical objects such as traffic lights during restoration. While our framework cannot recover detections in zero-proposal regions, it quantitatively characterizes flare interference as a proxy for local visual degradation. By providing this “awareness of blindness”, the system avoids falsely assuming a region is clear. Instead, it offers an explicit measure of visual uncertainty, allowing downstream fusion layers to dynamically prioritize stable sensors (LiDAR/Radar) in affected regions, thereby improving overall system safety.

## 6. Conclusions

Lens flare is a pervasive challenge in imaging systems, particularly for cameras utilized over extended durations. As autonomous driving technology becomes increasingly prevalent, the density of onboard cameras is expected to rise. Consequently, the impact of lens flare is likely to become more pronounced, potentially leading to a significant degradation in detection performance. However, this issue remains underrepresented in current public datasets, which are typically curated using pristine, well-maintained sensors. As a result, there has been limited research addressing the compounded effects of camera aging and lens flare on robust detection systems.

In this paper, we have presented a reference-free, lens-flare-aware detection framework to mitigate the adverse impact of lens flare on object detection. Compared to prior research, our approach not only provides a robust reference-free solution but also achieves a significant enhancement in AP through an end-to-end training pipeline and a teacher–student learning framework. Unlike existing methods and traditional lens flare removal models, our framework facilitates real-time processing, rendering it highly suitable for deployment in practical autonomous driving scenarios.

## Figures and Tables

**Table 1:** AP in BDD100K dataset with added lens flare.

Class	Original Object Detector (YOLOv5m) [19]	Reference-Based Model	Reference-Free Model
car	0.643 ± 0.004	0.659 ± 0.005	0.653 ± 0.004
traffic light	0.519 ± 0.006	0.543 ± 0.006	0.540 ± 0.006
traffic sign	0.535 ± 0.005	0.549 ± 0.003	0.547 ± 0.004
bicycle	0.357 ± 0.017	0.390 ± 0.035	0.380 ± 0.028
pedestrian	0.440 ± 0.004	0.457 ± 0.003	0.452 ± 0.003
truck	0.418 ± 0.006	0.437 ± 0.011	0.434 ± 0.009
bus	0.430 ± 0.014	0.450 ± 0.017	0.441 ± 0.014
motorcycle	0.277 ± 0.010	0.282 ± 0.008	0.281 ± 0.007
rider	0.302 ± 0.017	0.311 ± 0.017	0.308 ± 0.015

^1^ In this table, the reference-based and reference-free models denote the retrained LPIPS and ResNet-18 models, respectively. ^2^ Both our proposed reference-based and reference-free methods outperform the baseline object detector across all classes. Notably, although the reference-free model utilizes limited information from a single input, it still surpasses the original detector by a significant margin. ^3^ The performance gain of our proposed method depends on class-specific features and the number of detections; for classes with limited samples, such as “motorcycle”, the improvement is relatively modest.

**Table 2:** AP on cars for De-flare model.

	Lens-Flare-Corrupted Images	De-Flare Images [8]
AP	0.641	0.680

^1^ The detection is performed by YOLOv5m [19]. ^2^ We evaluate the de-flare model on the entire dataset ($6\times 10^{4}$ images). ^3^ Note that the performance of the de-flare model should be interpreted as “boosted” because the lens flares used in our experiments overlap with its training dataset. As illustrated in Figure 12, this model occasionally fails to generalize to real-world lens flares and may erroneously alter or remove traffic lights.

**Table 3:** Model size and efficiency.

Model	Inference Time (ms/Image) ^1^	Params (M) ^2^	MACs (M) ^2^
De-flare model [8] (Transformer-based)	191.44	20.45	322,758.31 ^3^
De-flare model [13] (CNN-based)	201.23	3.64	4,391,958.01 ^3^
Reference-free model	19.09	11.51	595.76
Reference-based model	18.73	2.47	393.02

^1^ To simulate a practical autonomous driving pipeline, we measure core inference time excluding I/O operations. The primary overhead in our method stems from cropping detection box patches from full images; notably, LPIPS/ResNet-18 and MLP inference account for only ∼10% of the total processing time. Our method’s low GPU footprint per category further enables parallel execution across multiple classes. For convenience, patches were cropped for all detected categories, but predictions were performed exclusively for the “car” class, which has the most instances in our dataset. ^2^ The Params and MACs were obtained using the THOP python tool. ^3^ For the de-flare model [8,13], as the input resolution exceeds 520×520, each image is processed in two separate crops, necessitating two model passes. Consequently, the reported MACs are doubled.

**Table 4:** Ablation results ^1^.

Lens Flare Perception		LLR Prediction		AP on Cars
MSD	LPIPS	MLP	KDE	
✔ ^2^			✔	0.646 ± 0.003
✔		✔		0.648 ± 0.003
	✔ ^3^		✔	0.647 ± 0.003
	✔	✔		**0.659 ± 0.005 ^4^**

^1^ MSD and LPIPS are utilized to estimate $m_{k}$, while MLP and KDE are employed for predicting LLR values. ^2^ ✔ denotes that the specific strategy or feature was employed in the experiment. ^3^ When using LPIPS with KDE, retraining the LPIPS model is not feasible because gradients cannot be backpropagated through the KDE. Thus, the pre-trained weights from [38] were adopted. ^4^ Bold values represent the highest results for the metric.

**Table 5:** Performance enhancement across diverse SOTA object detection architectures.

Model	Baseline AP	+Ours (Ref-Based)	+Ours (Ref-Free)
YOLOv10-m [42]	0.456 ± 0.002	0.470 ± 0.007	0.470 ± 0.007
YOLOv11-m [43]	0.467 ± 0.002	0.489 ± 0.007	0.488 ± 0.006
YOLOv12-m [44]	0.460 ± 0.001	0.480 ± 0.005	0.479 ± 0.004
RT-DETR-l [45]	0.468 ± 0.004	0.504 ± 0.010	0.504 ± 0.009

^1^ All detectors utilized weights pre-trained on the COCO dataset without further fine-tuning. ^2^ The lens flare perception and LLR prediction modules were retrained for each detector to account for their distinct behavioral patterns and error distributions.

**Table 6:** Performance comparison on real-world dataset.

Model	Baseline AP	+Ours (Ref-Free)
YOLOv10-m [42]	0.659	0.668
YOLOv11-m [43]	0.657	0.658
YOLOv12-m [44]	0.663	0.674
RT-DETR-l [45]	0.679	0.705

^1^ All detectors utilized weights pre-trained on the COCO dataset without further fine-tuning. ^2^ The lens flare perception and LLR prediction modules were obtained from Section 5.4.

## Data Availability

The data and code can be requested by contacting the corresponding author.
